# Using Super-Resolution for Enhancing Visual Perception and Segmentation Performance in Veterinary Cytology

**DOI:** 10.3390/life14030321

**Published:** 2024-02-28

**Authors:** Jakub Caputa, Maciej Wielgosz, Daria Łukasik, Paweł Russek, Jakub Grzeszczyk, Michał Karwatowski, Szymon Mazurek, Rafał Frączek, Anna Śmiech, Ernest Jamro, Sebastian Koryciak, Agnieszka Dąbrowska-Boruch, Marcin Pietroń, Kazimierz Wiatr

**Affiliations:** 1 ACC Cyfronet AGH, Nawojki 11, 30-950 Kraków, Poland; d.lukasik@cyfronet.pl (D.Ł.); russek@agh.edu.pl (P.R.); j.grzeszczyk@cyfronet.pl (J.G.); mkarwat@agh.edu.pl (M.K.); s.mazurek@cyfronet.pl (S.M.); rafalfr@agh.edu.pl (R.F.); jamro@agh.edu.pl (E.J.); koryciak@agh.edu.pl (S.K.); adabrow@agh.edu.pl (A.D.-B.); pietron@agh.edu.pl (M.P.); wiatr@agh.edu.pl (K.W.); 2 AGH University of Science and Technology, al. Mickiewicza 30, 30-059 Kraków, Poland; 3 University of Life Sciences, al. Akademicka 13, 20-950 Lublin, Poland; anna.smiech@up.lublin.pl

**Keywords:** super image resolution, computer vision, deep learning, cytology, medical imaging, semantic segmentation

## Abstract

The primary objective of this research was to enhance the quality of semantic segmentation in cytology images by incorporating super-resolution (SR) architectures. An additional contribution was the development of a novel dataset aimed at improving imaging quality in the presence of inaccurate focus. Our experimental results demonstrate that the integration of SR techniques into the segmentation pipeline can lead to a significant improvement of up to 25% in the mean average precision (mAP) metric. These findings suggest that leveraging SR architectures holds great promise for advancing the state-of-the-art in cytology image analysis.

## 1. Introduction, Motivation

Veterinary cytology is a specialized field within veterinary medicine that focuses on the microscopic examination of cells and cellular structures in animals. This discipline plays a crucial role in diagnosing and monitoring various diseases and conditions in animals. By analyzing the morphology, size, and arrangement of cells obtained through techniques like fine-needle aspiration, veterinarians can identify abnormalities such as cancerous growths or infections, aiding in the development of appropriate treatment plans.

The process of veterinary cytology can be time consuming and labor intensive, requiring skilled professionals to examine numerous cell samples under a microscope. Artificial intelligence can significantly expedite this process by automating cell image analysis, identifying and categorizing abnormalities, and providing veterinarians with quicker, more accurate diagnostic results. This not only saves valuable time but also enhances the efficiency of veterinary care, ultimately improving the outcomes for animals in need of diagnosis and treatment.

In recent years, deep learning-based solutions have emerged as a prominent topic in the field of Information Technology. Novel approaches are being developed and implemented daily to optimize, enhance, and facilitate various aspects of our lives. This growing trend is also evident in the medical domain, including veterinary medicine. It is important to note that deploying models for healthcare applications entails significant responsibility and necessitates rigorous testing and monitoring to mitigate any risks associated with artificial intelligence (AI) predictions.

Our research team is dedicated to developing solutions that assist veterinarians in making faster and more accurate diagnoses for their animal patients. Our prior work has focused on age classification [1], object segmentation [2], and detection [3] in the context of cytology imaging for canines. In this study, we continue our investigation into AI applications in the veterinary field, specifically exploring the combination of Super-Resolution (SR) and Semantic Segmentation techniques. By building upon previous research, we aim to further advance the state-of-the-art in veterinary image analysis and improve diagnostic outcomes.

## 2. State-of-the-Art, Reason for Conducting the Research

The acquisition of cytology images is a multifaceted process that involves the preparation of tissue samples using staining methods such as Diff-Quik, followed by the selection of suitable areas by a veterinary expert and image capture via a microscope-mounted camera. This study aims to address the challenges associated with obtaining images of inadequate focus or suboptimal quality for examination purposes. The integration of super-resolution (SR) techniques into the image analysis process represents a promising avenue for enhancing the quality and utility of cytology images, thereby facilitating more accurate and reliable diagnostics.

Research Questions:

Can deep learning-based architectures enhance the quality and resolution of cytology images, thereby facilitating improved image quality assessments? To what extent can such enhancements aid pathologists in diagnosing challenging or average-quality cases? Does the improvement of image quality augment the performance of semantic segmentation architectures in detecting carcinogenic cells within preparations?

### 2.1. Advancements in Super-Resolution and Semantic Segmentation

The field of medical imaging has seen significant advancements through the integration of deep learning techniques, particularly in super-resolution (SR) and semantic segmentation. He et al.’s development of deep residual learning for image recognition [4] laid the groundwork for subsequent innovations in image analysis, including the application of SR to improve image quality. Similarly, the introduction of U-Net by Ronneberger et al. [5] revolutionized biomedical image segmentation, offering a powerful tool for detailed tissue and cell analysis.

### 2.2. Dual Super-Resolution Learning for Semantic Segmentation

Building on these foundations, Wang et al. [6] proposed a dual super-resolution learning framework specifically designed to enhance segmentation accuracy without additional computational costs. This approach directly addresses the challenges faced in cytology image analysis, where the precision of segmentation is paramount. By integrating SR techniques into the segmentation process, this method not only improves image quality but also enhances the efficiency of diagnostic models.

### 2.3. Effectiveness of Super-Resolution in Medical Imaging

The effectiveness of SR techniques in medical imaging has been further demonstrated by Ledig et al. [7], who developed a generative adversarial network for photo-realistic super-resolution. This advancement underscores the potential of SR to significantly improve the resolution and clarity of medical images, facilitating more accurate diagnoses. Moreover, Dong et al. [8], Zhang et al. [9], Pereira et al. [10], Pham et al. [11], Goodfellow et al. [12] and Wang et al. [13] have contributed to the field by developing deep learning models that enhance the resolution of images, proving essential for detailed medical analysis and diagnosis.

### 2.4. Super-Resolution for Enhanced Diagnostic Accuracy

The integration of SR with semantic segmentation has shown promise in enhancing diagnostic accuracy. Studies by Chen et al. [14] and Zhou et al. [15] have demonstrated the potential of deep-learning models to improve semantic segmentation, which, when combined with high-resolution images obtained through SR techniques, can significantly aid in the detection and classification of diseases. Furthermore, the work by Isola et al. [16] on image-to-image translation with conditional adversarial networks has opened new avenues for applying SR in medical imaging, suggesting that enhancing image quality can substantially improve the performance of segmentation models.

### 2.5. Recent Progress and Integrative Approaches in Super-Resolution

Recent studies have underscored the transformative impact of super-resolution techniques across various domains of medical imaging, offering novel perspectives and methodological advances. For instance, Wang et al. [17] introduced a smarter microscope system that leverages E-CNN-based super-resolution to enhance single cell analysis, marking a significant step forward in cytology imaging. Similarly, Zhang et al. [18] explored the diagnostic potential of super-resolution in visualizing biliary structures, demonstrating its utility in clinical settings. Caputa et al. [19] specifically addressed veterinary cytology by integrating super-resolution to improve visual perception and segmentation performance, which is directly relevant to our research objectives. These studies, alongside contributions from Li et al. [20], Ma et al. [21], and Yuqian et al. [22], illustrate a collective move towards enhancing diagnostic accuracy and image quality through advanced computational techniques. The synthesis of super-resolution and deep learning not only promises to refine diagnostic capabilities in cytology but also sets a new benchmark for precision in medical image analysis.

## 3. Contribution, New Algorithm, Constructed System

**Figure 1:**
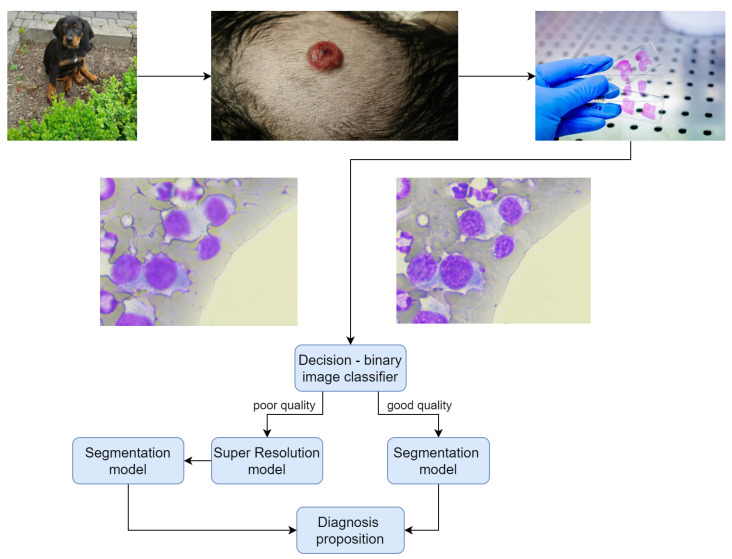
Proposed working system scheme.

The primary contribution of this study is the incorporation of a Super-Resolution module into the machine-learning pipeline, with the aim of enhancing the accuracy of segmentation models (Figure 1). This potential application for improving image quality emerged as a result of various distortions that may occur during the acquisition of cytological preparation images (Table 1). Our research is specifically focused on addressing poor sharpness distortions.

The objective of this study is to develop a machine learning model capable of enhancing the quality of images affected by improper focus settings on the microscope’s adjustment knob. To evaluate the effectiveness of this approach, the enhanced images will be compared to properly created images. The development of a dedicated dataset is a prerequisite for this evaluation [23].

In the context of veterinary examinations, an animal patient undergoes evaluation when visible skin alterations are observed. A tissue sample is subsequently obtained and examined under a microscope, during which an image is generated. In instances where the image quality is sub optimal or the microscope lens focus is improperly set, the decision block (binary image classifier) routes the image to the Super-Resolution model. Following this enhancement process, the segmentation model identifies objects within the image, and a diagnosis is proposed.

### 3.1. Novel Dataset

The majority of datasets for Super-Resolution (SR) tasks are artificially generated, employing image downscaling and interpolation techniques. However, the nature of the distortion we aim to address is distinct from these methods. Recognizing this led to the development of an experimental dataset [23] in collaboration with a veterinary expert, which is elaborated upon in the subsequent chapter.

The following algorithm was proposed for the acquisition of samples:

1. Identify the diagnostic region within the cytological preparation.
2. Adjust the microscope lens focus to obtain a high-quality image.
3. Intentionally alter the microscope’s adjustment knob to degrade the image quality and sharpness, thereby simulating the real-world distortion.

**Figure 2:**
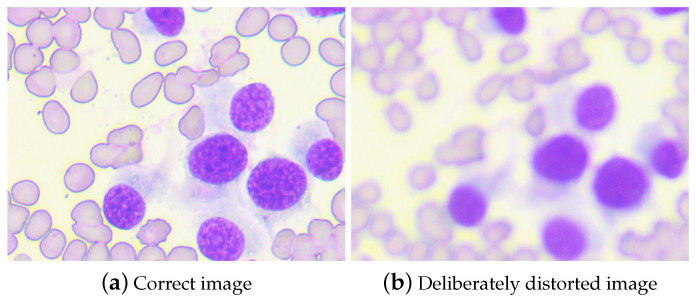
Examples of high and low sharpness images from the dataset. Magnification 400×.

This approach enabled the generation of a dataset that more accurately represents the specific type of distortion we aim to mitigate, providing a more suitable foundation for model training and evaluation as in Figure 2.

Following this procedure we collected 1192 high resolution (2592 × 1944) samples with their corresponding distorted versions.

### 3.2. Proposed New Super-Resolution Metric

**Figure A1:**
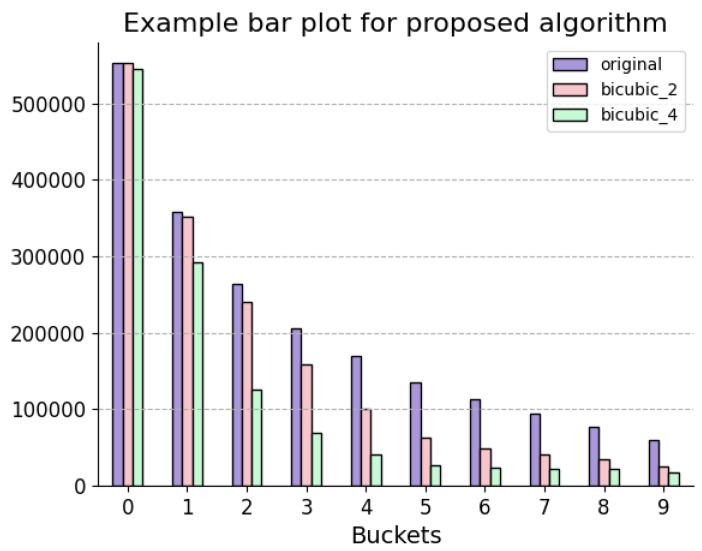
2D-DFT magnitude bar plot for original image and two bicubic interpolation degradations, example with 10 ring-shaped masks.

In this study, an additional metric based on frequency analysis is employed as a novel approach to assess segmentation performance. This method involves grouping the energy computed using a two-dimensional Discrete Fourier Transform (DFT-2D) by frequency. This operation enables the observation of the total energy within each frequency range, providing insights into how machine learning models affect high frequencies in the image, which contribute to visual sharpness. The procedure for this approach is as follows (Figure A1):

1. Open an image in YCbCr mode and use only the luminance channel.
2. Compute the two-dimensional discrete Fourier Transform and shift the zero-frequency component to the center of the spectrum.
3. Calculate the absolute sum of all magnitudes for a chosen set of ring-shaped masks and display the results in a bar plot.

This method employs visual representation to analyze the frequencies present within an image. Primary advantage is that it is not based on singular metrics such as SSIM [24] or LPIPS [25], as it captures changes in distribution subsequent to the application of filters or models.

### 3.3. Research Formula for Specific Medical Use Case with Unknown Degradation

During the course of the research, addressing the super-resolution (SR) task when the nature of degradation is unknown was a significant concern. The following strategies were assessed:

1. Use pre-trained models on various datasets;
2. Investigate known degradations, such as bicubic interpolation on our medical dataset;
3. Develop a dedicated super-resolution dataset exhibiting the same degradation intended to be mitigated.

The most favorable results were achieved using the third approach; however, it is crucial to consider feedback from domain experts in the medical field. It was discovered that applying super-resolution to images introduced artifacts that would not typically be present in cytology images. Veterinary specialists tended to favor lower-quality images over sharper ones, as the artifacts introduced by SR models hindered the diagnostic process. One of the key conclusions drawn is that a sharper image does not necessarily equate to a superior model. Therefore, in medical applications metrics cannot be the only evaluator for performance assessment.

## 4. Experiments and Results

This chapter provides a detailed look at the experiments conducted during this research study. Each subchapter explains the different stages of the study in a more accessible way, while still maintaining scientific accuracy.

### 4.1. Comparison of Possible Distortions in Cytology Imaging

Table 1 presents a list of potential distortions that may occur during image creation by veterinary experts. These hypothetical scenarios may require the application of Super-Resolution models as a preprocessing step to recover the images to their desired quality.

### 4.2. Comparison of Different Image Upsampling Methods Using Scale Factor 2

**Figure A2:**
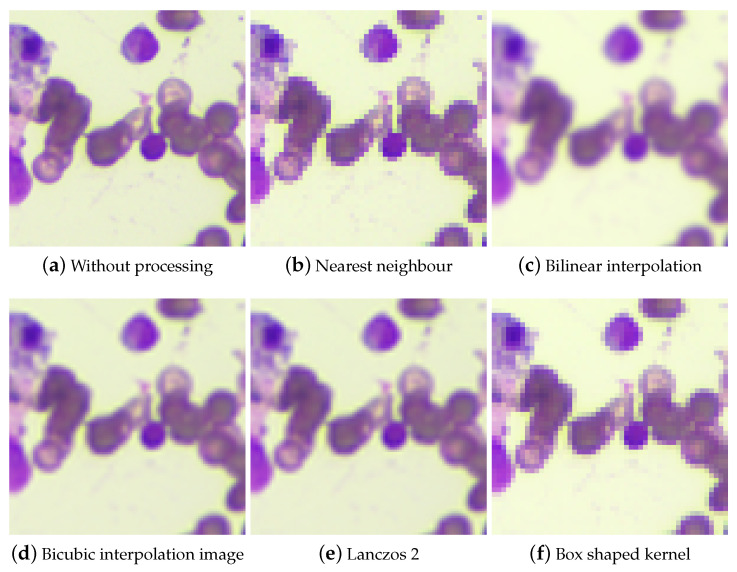
Comparison of different image upsampling methods using Scale 2. Magnification 400×.

This section presents a visual analysis of various classical image upsampling techniques when applied with a scale factor of 2. The objective is to evaluate the performance of each method in terms of image quality, preservation of structural details, and overall effectiveness in enhancing the resolution of the original image (Figure A2).

### 4.3. Pretrained Segmentation Model Approach

This section contains the results of inferencing pretrained Super-Resolution models and measuring their impact on semantic segmentation task on our cytology dataset.

In this comparative analysis, three state-of-the-art image upsampling architectures were selected for evaluation: SwinIR, BSRGAN, and RealSRGAN. The goal was to assess each model’s performance in terms of image quality and impact on segmentation metrics.

For segmentation evaluation, a deep learning model based on the Cascade Mask R-CNN [26] architecture was selected. The ResNeSt101 [27], which employs skip connections (i.e., input values bypass the current layer without any modifications, and are then summed with the modified input), was used for feature extraction. The model was initialized with weights pre-trained on the MS COCO dataset [28] and subsequently fine-tuned on cytology images.

#### 4.3.1. Impact of Bicubic Interpolation on Segmentation Inference

**Figure A3:**
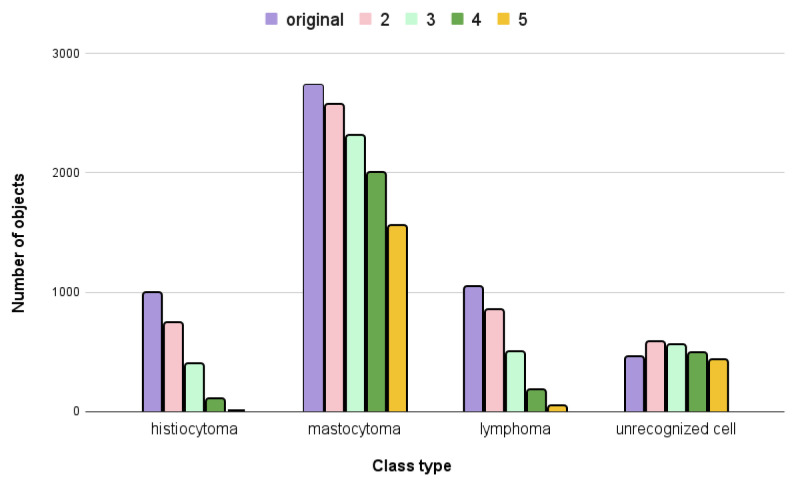
True Positive cell detection numbers for different scaling factors.

The results presented in Table 2 reveal an expected trend. As the bicubic interpolation scaling factor increases, which corresponds to a greater loss of information, both segmentation and super-resolution metrics are negatively affected. A higher scaling factor leads to increased confusion between objects. For instance, with bicubic interpolation using a scaling factor of five, almost no cells are accurately recognized for the two cancer types, as illustrated in Figure A3.

**Figure A4:**
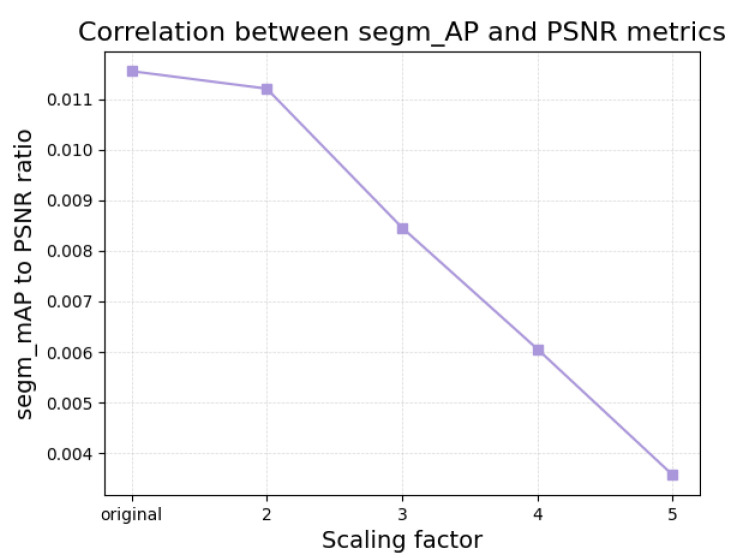
Correlation between segmAP and PSNR metrics for different scaling factors.

Figure A4 presents the relationship between the segmentation Average Precision (segmAP) and Peak Signal-to-Noise Ratio (PSNR) metrics. A decreasing trend in the ratio between these two metrics is observed when the scaling factor increases. This indicates that, in some cases, a linear correlation exists between the performance metrics of these two distinct computer vision tasks. As the scaling factor increases, the quality of the image and segmentation performance both tend to degrade.

#### 4.3.2. Impact of the Pre-Trained Super-Resolution Models on Segmentation Inference

In this phase, a comparative analysis is conducted to evaluate the performance of the segmentation model on the original dataset opposite to bicubic interpolation with scaling factors of two and five. The primary objective is to investigate whether employing pre-trained models can enhance the accuracy of the segmentation process.

The application of various super-resolution (SR) architectures on the original data did not yield any improvements in segmentation quality. Nevertheless, the minimal loss in mean average precision (mAP) indicates that the model is proficient in identifying cancer cells that have undergone enhancement through the SR process (Table 3).

When employing image enhancement techniques for damaged data with decimation and bicubic interpolation, the results, as presented in Table A1 and Table A2, are found to be worse. This suggests that the utilization of SR introduces additional noise to the data, consequently leading to poorer performance by the segmentation model. This outcome was anticipated, given that the model was pre-trained on original data and was subsequently required to handle processed data during the inference phase.

**Figure A5:**
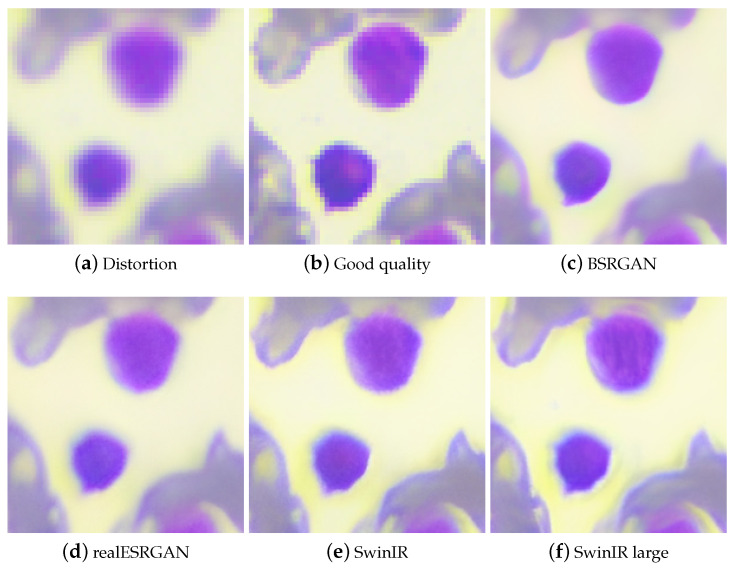
Example results of chosen architectures (cropped part of the image). Magnification 400×.

The findings indicate that the naive application of SR models to images does not yield improvements in segmentation quality. However, it does enhance the perceptual quality of the image, as depicted in Figure A5. In certain instances, it also results in an increase in SR metrics, as demonstrated in Table 3.

The obtained results were subsequently reviewed in consultation with a veterinary expert. It was determined that, although the images appeared sharper following the application of the super-resolution model, the presence of certain artifacts rendered them less reliable than the original images. The enhanced image quality did not contribute to improved diagnostic accuracy, as these artifacts introduced elements that would not typically be found in cytology images.

#### 4.3.3. Training Semantic Segmentation Model on Super-Resolution Medical Data

In this experiment, we trained and tested the segmentation on data processed in various ways. As demonstrated in Table 4, we downsampled the images using decimation and bicubic interpolation and then upsampled using super-resolution architectures. While this research may not hold practical significance from a medical standpoint, as manipulating original data is generally discouraged, it does reveal that the optimal results for cancer cell recognition are obtained when utilizing undistorted, original data.

#### 4.3.4. Dedicated Dataset Experiments for Super-Resolution

Ultimately, the experiments were carried out on a dedicated dataset, with the SwinIR architecture selected for training [29].

During the exploration of the dataset, the data distribution was analyzed. The histograms presented in Figure A8 showcase the PSNR values for both bicubic interpolation and our dedicated dataset, in comparison to high-resolution original data. The spectrum of our dataset is wider and there are images that would be considered of a good quality in terms of pixel loss. In contrast, bicubic interpolation exhibits less diversity, limiting its applicability to the restoration of specific distortion types.

The dedicated dataset encompasses various forms of degradation that are likely to be encountered in cytology images.

**Figure A6:**
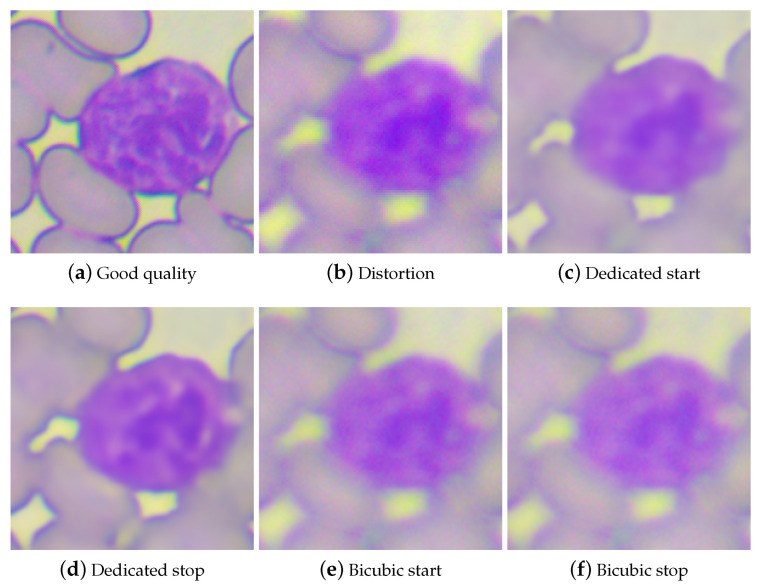
Comparison of results for training on dedicated and bicubic datasets. Magnification 400×.

The initial experiment exhibited a substantial improvement in the PSNR metric upon training the SwinIR model on our dataset, as displayed in Table A3. This improvement is also evident in the inferred images after training, presented in Figure A6.

The transformer model underwent training for approximately 1000 epochs, utilizing four NVIDIA V-100 GPUs from the ACK Cyfronet Prometheus supercomputer [30]. Default parameters tailored for the classical super-resolution task were employed. The upsampling factor was set to 2.

Experiments involving training on our dataset demonstrated a notable improvement when compared to the bicubic dataset, which is commonly utilized in super-resolution tasks.

**Figure A7:**
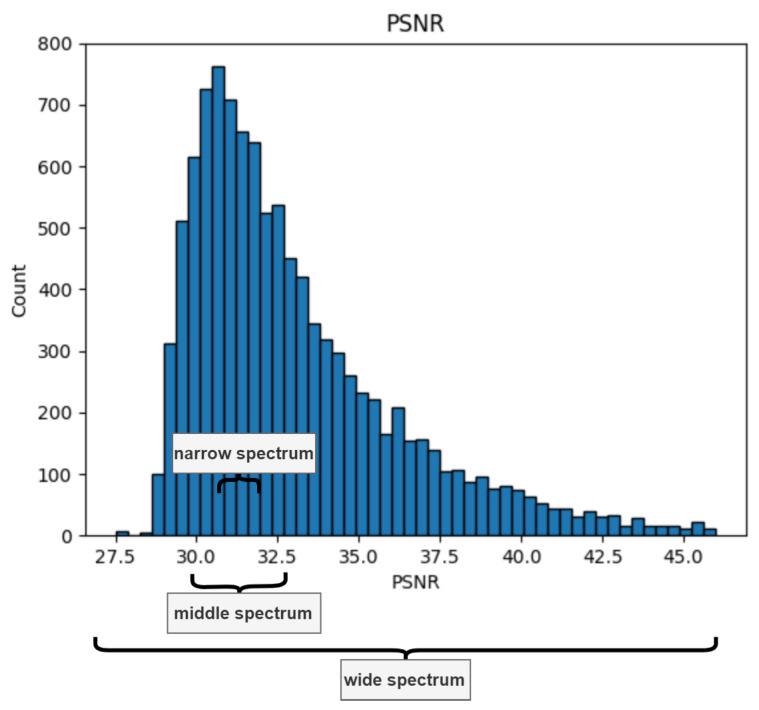
Histogram presenting chosen spectrums.

**Figure A8:**
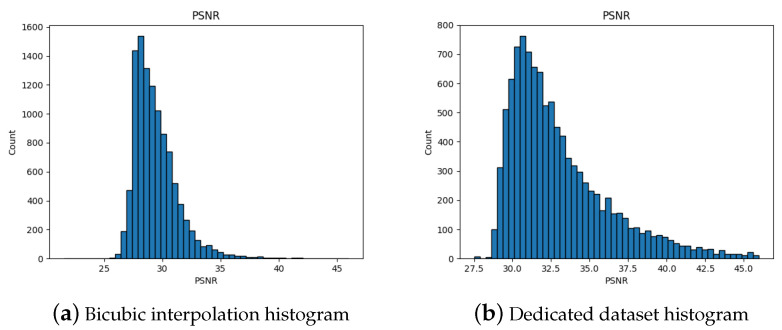
Comparison of histograms for datasets.

The second experiment focused on examining the influence of the selected spectrum. We investigated whether a narrow or wide spectrum of our dataset would yield superior results. Figure A7 illustrates the three distinct segments of the dataset that were employed in this experiment.

**Figure 3:**
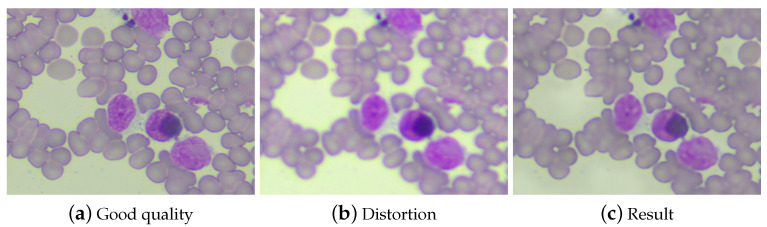
Example result for training on a wide spectrum. Magnification 400×.

The experiment reveals that training on the widest spectrum (as illustrated in Figure A7) leads to the the most favorable results for both narrow and wide spectrum test datasets, as presented in Table A4. This finding suggests that the model effectively learns to reconstruct images when the training data encompasses a diverse range of distortions with varying types and levels (Figure 3).

#### 4.3.5. Improving Segmentation Metrics Results with Super-Resolution

For end-to-end experiments aimed at enhancing segmentation using super-resolution (SR) architectures, a new subset of the dataset was employed. This subset comprised both semantic segmentation annotations and distorted images representing the exact same area.

The results depicted in the subsequent table exhibit promising outcomes. In certain methodologies, the results show improvements when compared to the low-quality dataset. For instance, with the BSRGAN architecture, employing a 4x upsampling technique followed by subsampling to the required resolution, the improvement reaches up to 25% when compared to the results that would have been obtained using a low-quality dataset. As anticipated, the most optimal results are achieved when training the segmentation on high-quality images, where the segm_mAP_75 increase is up to 64%. Interesting aspect of the following experiments is that using super-resolution as a first step, before subsampling to desired annotations size leads to better results than the opposite operation. This is because subsampling as a first step loses even more information that can not be restored during upsampling step, leading to lower segmentation results.

The intriguing outcome of the subsequent experiments in Table 5 suggests that implementing super-resolution as an initial step, prior to the subsampling process to achieve the desired annotation size, yields superior results compared to the inverse sequence of operations. This observed phenomena could be primarily attributed to the fact that the adoption of subsampling as a preliminary step entails an excessive loss of information. This loss of detail, once occurred, is unable to be fully recuperated during the upsampling process, culminating in compromised segmentation outcomes. Hence, the order of these processing steps plays a critical role in maximizing the data fidelity and overall accuracy of the segmentation results.

## 5. Conclusions

The presented research, which encompasses two fields of Computer Vision—Super-Resolution and Semantic Segmentation—underscores the possibility of enhancing the quality of medical images for their interpretation and analysis. The primary challenges identified during the investigation include a scarcity of data and differing perceptions between programmers and medical experts.

The first challenge stems from the nature of the formulated problem. Restoring an image from an unknown degradation is a daunting task, particularly in medical imaging. Consequently, a unique dataset was created for this study, enabling improvements in both super-resolution metrics and human perception. The second challenge arises from the specific use case provided; veterinary experts analyze medical images differently from individuals unfamiliar with cytology. The realization that sharper, high-resolution images are not always preferable for diagnosis due to artifacts observable after applying AI models was not immediately evident.

This research primary achievements include formulating a potential procedure for addressing unknown degradation, such as incorrectly set sharpness on a microscope. The steps taken during the experimental phase could potentially be applied to other domain-specific use cases. Another valuable aspect is the identification and analysis of possible distortions in medical imaging. We facilitated a better understanding of the problem’s nature and its uniqueness compared to standard Super-Resolution tasks.

Finally as expected, the substantial increase in the PSNR measure during SwinIR architecture training (Table A3) and the visual perception improvement shown in Figure A6 are noteworthy. The remarkable improvement of up to 25% in certain experimental scenarios, with respect to the segm_mAP metric change, is also worth mentioning. This underscores the potential of the applied methods in enhancing the performance of image segmentation tasks in medical imaging.

## Figures and Tables

**Table 1:** Potential distortions in veterinary image creation.

Distortion Type	Image	Description
correct image	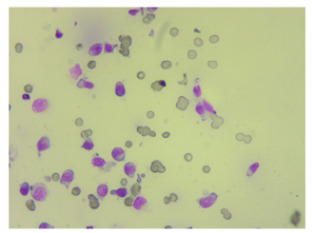	Image properly created
dark lighting	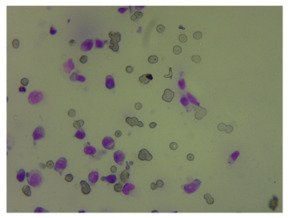	microscope bulb is not turned on or the room where the image is created is dark, the resulting image may suffer from low contrast and poor illumination.
closed aperture	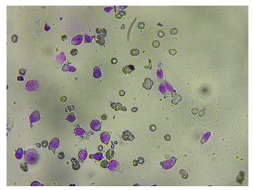	Responsible for the amount of the light that comes to a focus in the image plane
closed condensor	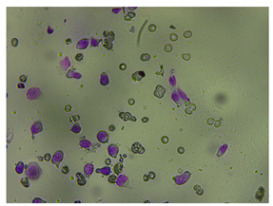	An improperly adjusted condenser, which is responsible for providing evenly distributed illumination
dark outside	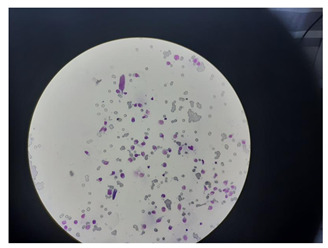	The image was not directly at the lens leading to dark edges
bad sharpness	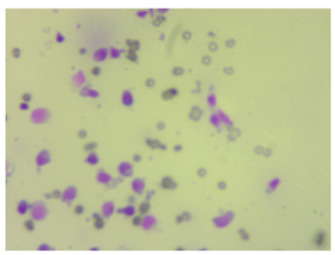	The microscope screw set inaccurately or the focus is set for the background of the image

**Table 2:** Comparison of super-resolution and segmentation metrics using pretrained segmentation model for inference.

Factor	Segm_mAP	Avg_Precision	Avg Recall	PSNR	SSIM	LPIPS
Original	0.439	0.623	0.679	-	1	0
2	0.392	0.598	0.663	34.98	0.93	0.07
3	0.276	0.492	0.573	32.62	0.82	0.17
4	0.195	0.444	0.540	32.19	0.79	0.26
5	0.113	0.348	0.456	31.68	0.74	0.35

**Table 3:** Results for inference on the test set for the original dataset.

Data Set Original	Segm_mAP	Avg_Precision	Avg_Recall	PSNR	SSIM	LPIPS
Original	0.457	0.596	0.654	-	-	-
RealSRGAN	0.446	0.587	0.540	36.16	0.96	0.050
BSRGAN	0.430	0.572	0.636	33.54	0.93	0.092
SwinIR	0.421	0.567	0.630	35.10	0.96	0.057
SwinIR_large	0.457	0.584	0.643	37.18	0.977	0.041

**Table 4:** Training semantic segmentation on interpolated and super-resolution data.

Data Set	Data Set Explanation	Segm_mAP	Avg_Precision	Avg_Recall
original	Original cancer inflammation dataset	**0.494**	**0.494**	0.584
bicubic2	Original dataset decimated and interpolated using scaling factor two (half of the pixels left after decimation)	0.465	0.465	0.567
bicubic4	Original dataset decimated and interpolated using scaling factor four (quarter of the pixels left after decimation)	0.423	0.423	0.527
realSRGAN ×4	Original dataset upscaled using realSRGAN and resized to original size	0.487	0.487	**0.587**
BSRGAN ×4	Original dataset upscaled using BSRGAM and resized to original size	0.478	0.478	0.587
SwinIR ×4	Original dataset upscaled using SwinIR and resized to original size	0.482	0.482	0.579

**Table 5:** End-to-end pipeline tests BSRGAN.

Experiment Summary	Segm_ mAP_50	Segm_ mAP_75	Segm_mAP_50 Percent_Change	Segm_mAP_75 Percent_Change
high quality dataset	**0.314**	**0.255**	38.94%	63.46%
low quality dataset	0.226	0.156	0.00%	0.00%
subsampling ×2, SR ×2	0.198	0.120	−12.39%	−23.08%
SR ×2, subsampling ×2	0.233	0.110	3.10%	−29.49%
subsampling ×4, SR ×4	0.227	0.155	0.44%	−0.64%
SR ×4, subsampling ×4	**0.279**	**0.195**	23.45%	25.00%

## Data Availability

Data available in a publicly accessible repository. The data presented in this study are openly available at https://github.com/jakubcaputa/super-resolution-dataset (accessed on 22 February 2024).

## Appendix A

**Table A1:** Results for inference on the test set for bicubic interpolation with Factor 2.

Data Set Bicubic2	Segm_mAP	Avg_Precision	Avg Recall	PSNR	SSIM	LPIPS
**original**	**0.426**	**0.566**	**0.633**	**34.69**	**0.92**	0.0084
realSRGAN	0.379	0.559	0.624	34.49	0.92	**0.073**
BSRGAN	0.360	0.536	0.607	33.77	0.90	0.090
SwinIR	0.345	0.534	0.606	33.54	0.91	0.087
SwinIR_large	0.339	0.541	0.613	33.70	0.91	0.091

**Table A2:** Results for inference on the test set for bicubic interpolation with Factor 5.

Data Set Bicubic5	Segm_mAP	Avg_Precision	Avg Recall	PSNR	SSIM	LPIPS
**original**	**0.116**	**0.311**	**0.419**	**31.58**	**0.72**	**0.377**
realSRGAN	0.092	0.300	0.397	31.38	0.69	0.252
BSRGAN	0.093	0.276	0.381	31.46	0.70	0.282
SwinIR	0.071	0.250	0.374	31.29	0.69	0.301
SwinIR_large	0.055	0.227	0.357	31.17	0.67	0.348

**Table A3:** Dedicated and bicubic results.

Train Data Set	Test Data Set	PSNR
bicubic	dedicated	25.84
dedicated	dedicated	**29.99**

**Table A4:** Spectrum experiments PSNR value.

Trained				
**Validated**	**Spectrum**	**Narrow**	**Middle**	**Wide**
	**Narrow**	30.41	**30.14**	29.73
	**Middle**	30.64	30.06	29.69
	**Wide**	**30.76**	29.40	**29.90**
